# In Situ Reactive Formation of Mixed Oxides in Additively Manufactured Cobalt Alloy

**DOI:** 10.3390/ma16103707

**Published:** 2023-05-13

**Authors:** Jack Lopez, Rok Cerne, David Ho, Devin Madigan, Qing Shen, Bo Yang, Joseph Corpus, William Jarosinski, Haiyan Wang, Xinghang Zhang

**Affiliations:** 1 School of Materials Engineering, Purdue University, West Lafayette, IN 47907, USA; lopez86@purdue.edu (J.L.);; 2 Praxair Surface Technologies, Inc., Indianapolis, IN 46222, USA; 3 School of Electrical and Computer Engineering, Purdue University, West Lafayette, IN 47907, USA

**Keywords:** additive manufacturing, laser powder bed fusion, oxide dispersion strengthening, Orowan strengthening, reduction–oxidation reaction

## Abstract

Oxide-dispersion-strengthened (ODS) alloys have long been considered for high temperature turbine, spacecraft, and nuclear reactor components due to their high temperature strength and radiation resistance. Conventional synthesis approaches of ODS alloys involve ball milling of powders and consolidation. In this work, a process-synergistic approach is used to introduce oxide particles during laser powder bed fusion (LPBF). Chromium (III) oxide (Cr_2_O_3_) powders are blended with a cobalt-based alloy, Mar-M 509, and exposed to laser irradiation, resulting in reduction–oxidation reactions involving metal (Ta, Ti, Zr) ions from the metal matrix to form mixed oxides of increased thermodynamic stability. A microstructure analysis indicates the formation of nanoscale spherical mixed oxide particles as well as large agglomerates with internal cracks. Chemical analyses confirm the presence of Ta, Ti, and Zr in agglomerated oxides, but primarily Zr in the nanoscale oxides. Mechanical testing reveals that agglomerate particle cracking is detrimental to tensile ductility compared to the base alloy, suggesting the need for improved processing methods to break up oxide particle clusters and promote their uniform dispersion during laser exposure.

## 1. Introduction

Laser powder bed fusion (LPBF) is a widely used metal additive manufacturing (AM) technology, which has advanced beyond rapid prototyping and into commercial part production. For high-value-added aerospace and medical industries, the resolution and part complexity achievable with LPBF can lead to improved performance [1]. In fact, the strength of LPBF AM components often exceeds their cast or wrought counterparts, owing to a refined microstructure formed during the rapid laser melting and the resolidification of metal powders [2]. A further improvement of material performance for high temperature components can be achieved by metal matrix composites (MMCs) reinforced with high temperature ceramic particles [3]. An important type of MMCs is the oxide-dispersion-strengthened (ODS) alloy. It is challenging to use AM techniques to fabricate ODS alloys, and it is important to investigate discrete powder particle mixtures to identify and assess the processing challenges.

ODS MMCs are desirable for nuclear applications for their resistance to radiation-induced void swelling and high strength at high temperatures [4,5]. Since oxide particles are hard enough to be considered nondeformable, the dislocation bowing theory indicates that their strengthening effect is maximized at smaller particle radii [6]. Oxide particles act as stable pinning sites, preventing grain growth at higher temperatures. Traditional approaches for producing an ODS material involve long-duration mechanical alloying combined with powder metallurgy consolidation methods such as spark plasma sintering and hot isostatic pressing [7,8,9]. Another process called gas-atomized reactive (GAR) atomization has been employed to form oxide particles within metal powder particles by the controlled introduction of oxygen into the argon gas atomization process [10,11,12,13]. Similarly, some studies have formed the oxide particles during LPBF by the addition of oxygen to the process atmosphere [14,15]. This work uses a straightforward approach of the mixture of oxide nanopowders with LPBF alloy powders prior to LPBF fabrication, which has been shown to be capable of forming nanoscale oxide reinforcement [16,17].

While powder metallurgy approaches can achieve small oxide sizes, these methods pose challenges for the fabrication of complex part geometries. Consolidation techniques generally require simple geometry even for pressure distribution, and the fine crystallites produced in mechanical alloying make the deformation processing of complex shapes arduous. In contrast, LPBF can produce parts with complex geometric features, which do not add to the processing cost [18]. Additionally, the local laser melting of the tracks of powder material can also promote oxide-particle-size refinement and distribution [19]. In conjunction, oxide particles in the melt pool can act as nucleation sites, refining grain size and reducing grain texture anisotropy. Multiple groups have studied the use of LPBF as a fabrication method for particle-reinforced MMCs. In some cases, an oxygen-containing atmosphere is used for in situ oxide synthesis in the melt pool [19]. Directed energy deposition (DED), a laser-based powder-fed process with large melt pools has also been used [20]. Typical reinforcement particles include SiC, TiC, Y_2_O_3_, and Al_2_O_3_ [17,21,22,23,24] in matrix alloys such as Al, ferritic or austenitic steels, or Ni alloys [14]. In ODS with titanium-containing matrix alloys, a complex Y_2_Ti_2_O_7_ oxide is formed [25]. Similarly, multiple groups have shown that the addition of elemental hafnium results in the formation of a Y_2_Hf_2_O_7_ complex oxide accompanied with a decrease in oxide particle size from 50 nm to 10 nm [26,27].

To date, there is little study on Co-based ODS alloys. The Mar-M 509 cobalt alloy was introduced in 1965 by Lockheed Martin as an elevated temperature cast alloy strengthened with carbides for blading applications [28,29,30]. Cobalt has a higher melting temperature and higher thermal conductivity than Nickel, enabling higher operating temperatures in actively cooled components [31]. Co-base alloys such as Mar-M 509 also possess improved sulfidation resistance [32]. While in many of the hot sections of engines, where blades have been replaced with Ni-base superalloys due to the improved creep resistance mechanisms in γ/γ′ microstructures, there remains a need for carbide- and solid solution-strengthened Co alloys for stationary components such as nozzle guide vanes [33]. These components encounter high temperature exposure for long periods and applied loads are low enough that thermomechanical fatigue and surface oxidation become a more important concern [34]. Solid solution strengthening contributions arise from the matrix substitution of W, Ta, and Cr [35]. Carbides are desirable precipitates that improve tensile and creep strength and they have better thermal stability than γ′ precipitates, [36,37,38,39]. In cast Mar-M 509, Cr contributes to the formation of M_23_C_6_ carbides at room and intermediate temperatures and M_7_C_3_ carbides at higher temperatures during solidification [29,40]. Mixed MC carbides enriched in Ta, W, Ti, and Zr are stable across all temperatures and form in the mushy zone during solidification [40,41]. However, the exact carbide phases present from nonequilibrium solidification in LPBF Mar-M 509 are not well characterized.

In this work, 1.5 weight percent Cr_2_O_3_ powders were incorporated into Mar-M 509 Co alloy powders and processed into ODS composite material by LPBF. The resulting microstructures contained a combination of 10–100 nm oxide nanoparticles within cellular solidification structures and 10–50 µm oxide agglomerates, which are extensively characterized by microscopy. Both morphologies of oxide transformed into a mixed Ta-, Ti-, Zr-rich oxide phase by a hypothesized reduction–oxidation reaction. Tension testing and fracture surface investigation indicated that the larger agglomerated oxide particles are responsible for greatly reduced tensile strain. Both the nanoscale oxides and micron-scale agglomerates suggest that improvements in processing can lead to success in the fabrication of homogenous ODS alloys by LPBF.

## 2. Materials and Methods

**Figure 1:**
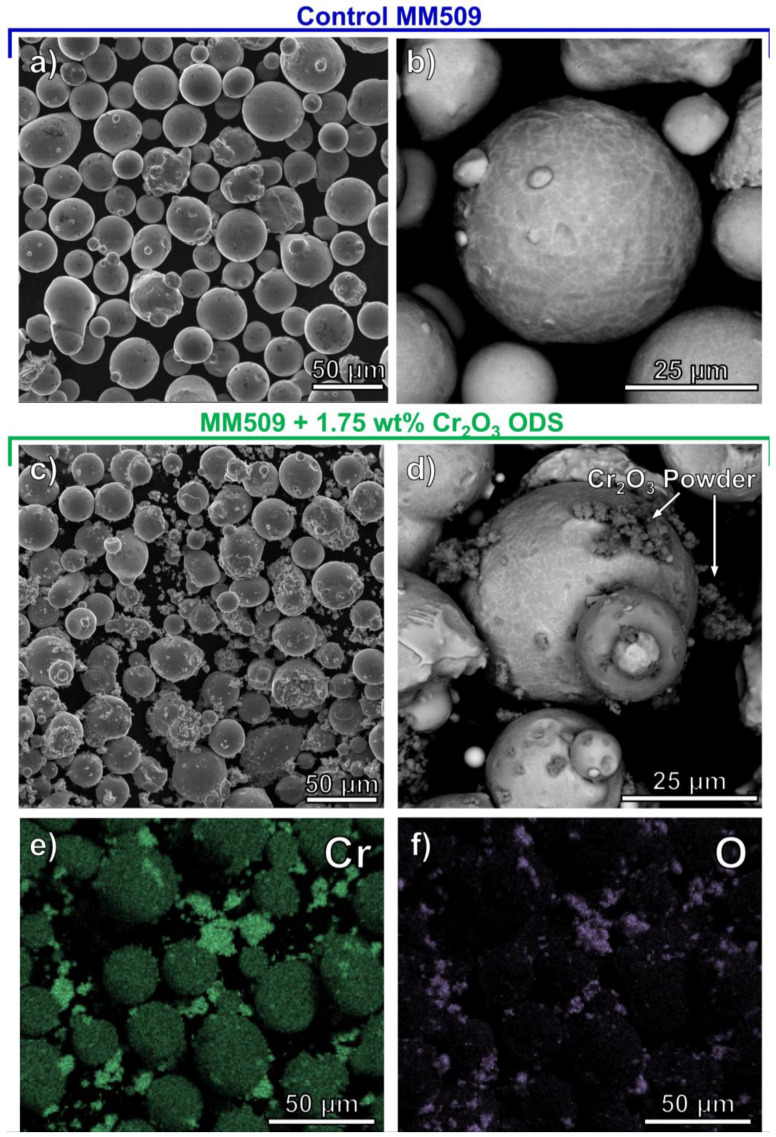
Powder morphology of (**a**,**b**) as-received (control) and (**c**–**f**) as-mixed 1.75 wt% Cr_2_O_3_ powder for LPBF. EDS results in (**e**,**f**) show the presence of micron-scale Cr_2_O_3_ on the surfaces of metallic particles as well as in isolated agglomerates.

LPBF specimens were produced using commercial Mar-M 509 argon gas-atomized powder sourced from Praxair Surface Technologies, Inc. (PST), Indianapolis, IN, USA. Scanning electron microscopy (SEM) images of the as-received powders are given in Figure 1a,b. The powder particle size was measured by MICROTRAC analysis, providing results of d_10_, d_50_, d_90_ (10th, 50th, 90th diameter percentile) of 22.2, 30.4, and 43.6 µm, respectively. The Mar-M 509 alloy powder chemistry as measured by inductively coupled plasma mass spectrometry is provided in Table 1. Cr_2_O_3_ powder provided by PST had MICROTRAC d_10_, d_50_, d_90_ of 1.59, 3.22, and 9.97 µm, respectively. ODS powders and Co alloy powders were mixed in a commercial V blender until uniform and stored in containers filled with argon gas until use in additive manufacturing. SEM micrographs in Figure 1 indicate that the Cr_2_O_3_ powder exists as surface-bonded and isolated forms, as indicated by Figure 1d and the energy dispersive spectroscopy (EDS) map in Figure 1e,f.

Both virgin and ODS Mar-M 509 powders were fabricated using an EOS M290 LPBF device on 316L stainless steel build plate held at 80 °C using baseline parameters of 285 W laser power, 960 mm/s laser scan speed, 110 µm hatch spacing, and 40 µm nominal layer thickness. Using Equation (1), the volumetric energy density (VED), *ψ*, can be calculated, which is a useful index for comparing relative heat input of combinations of parameter sets. *P* is the laser power in W, *v* is the laser scan speed in mm/s, *d* is the layer thickness in mm, resulting in *ψ* units of J/mm^3^.

(1) $$\psi =\frac{P}{v*h*d}$$

Laser power and scan speed were increased/decreased by 15% in conjunction with probe higher and lower energy inputs. The parameter sets and respective energy densities are given in Table 2. Sample geometries fabricated consisted of cubes with 1 cm^3^ volume for microstructure analysis and 40 × 8 × 1.5 mm flat coupons for tensile dogbone production, oriented with the 1.5 × 40 mm face bonded to the build plate. The LPBF scan strategy utilized a contour–infill approach where the infill is a simple raster across the part profile. The raster direction was rotated by 67° for each successive layer to prevent the alignment of laser track boundaries.

Fabricated samples were removed from the 316 stainless-steel build plate by wire electrical discharge machining. Tensile dogbones were laser-cut and polished to 800 grit SiC paper to reduce the impact of surface defects on tension results. Tension testing was performed using an MTS Insight 100 Universal Test System equipped with a 30 kN load cell and a strain rate of 10^−3^. Strain was measured using an extensometer.

X-ray diffraction was performed on the XY and YZ polished planes using a Bruker (Billerica, MA, USA) D8 Focus with Cu Kα radiation (λ = 1.54 Å) over the 2θ range from 30° to 100°. SEM and electron dispersive spectroscopy (EDS) were performed using FEI (Hillsboro, OH, USA) Quanta 650 Field Emission Gun (FEG) and FEI Quanta 3D FEG microscopes using 20 kV accelerating voltage.

Samples for transmission electron microscopy (TEM) were prepared by mechanically grinding foils to ~70 µm in thickness before using the twin-jet polishing method to form an electron-transparent region surrounding the electropolished perforation in the 3 mm punched disc. Twin-jet polishing was performed with a Fischione (Export, PA, USA) Model 110 electropolisher using a 5% perchloric acid solution in ethanol cooled to −30 °C at the beginning of polishing. A 27 V potential was applied, resulting in a current of 16 mA through the disc sample. TEM and EDS experiments were performed using an FEI Talos 200X transmission electron microscope operated at 200 kV.

## 3. Results

### 3.1. X-ray Diffraction and Texture Analysis

**Figure 2:**
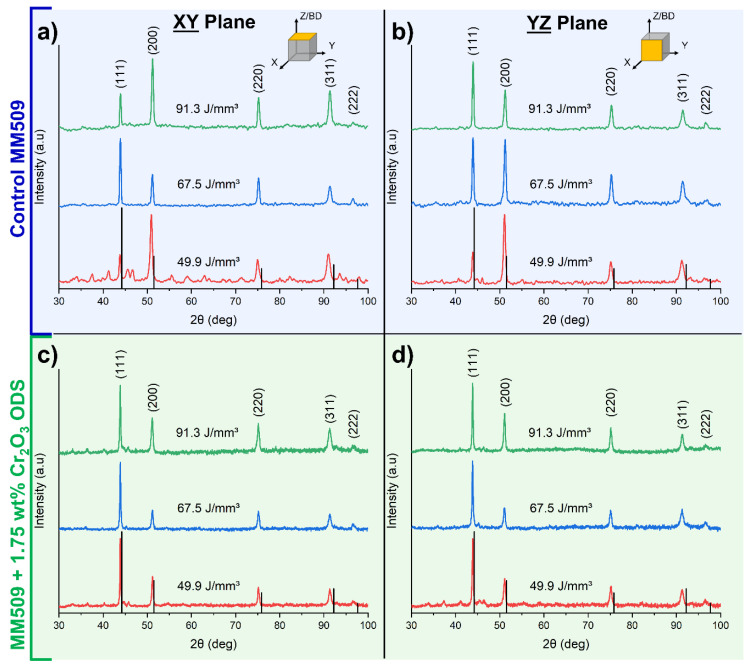
X-ray diffraction spectra for (**a**,**b**) control and (**c**,**d**) ODS samples printed with different parameters denoted by energy density. Peak shift compared to Co FCC reference peaks is observed. ODS samples have (111) predominant peaks compared to (200) texture in the control samples. Carbide peaks are detectable but weak in most samples.

The X-ray diffraction spectra for control and ODS materials in the XY and YZ planes can be seen in Figure 2. As has been previously observed in laser additive-manufactured microstructures with face-centered cubic (FCC) matrix crystal structures, there is preferential orientation along the (200) direction of the XY plane of control samples (Figure 2d) [42]. This is the preferred growth direction in FCC crystals during directional solidification due to thermal gradients [43]. The dominant peak in the ODS samples shifts to the (111) peak. Peak shift toward the lower 2θ compared to the reference peaks indicates a larger interplanar spacing in the Mar-M 509 alloy. This effect can be explained by the presence of solid solution elements in the matrix such as Cr, Ta, and W, which have larger atomic radii than the Co, increasing the interplanar spacing. Low intensity carbide peaks are visible in some samples, but the fluorescence phenomenon of cobalt under Cu Kα radiation causes high background intensity, making the reliable deconvolution of low intensity peaks from the background challenging.

### 3.2. Microstructure Analysis

**Figure 3:**
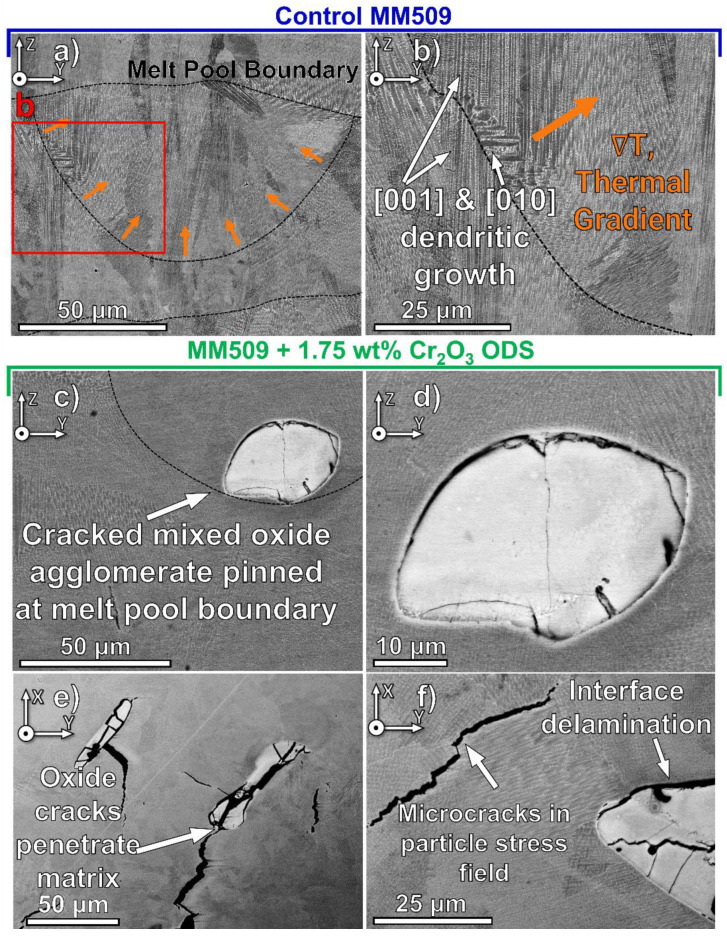
As-printed structure of (**a**,**b**) control and (**c**–**f**) ODS alloys displaying the cellular and dendritic solidification structures in YZ (**a**–**d**) and XY (**e**,**f**) planes. Subfigure (**b**) is a higher magnification of the box region in (**a**). Internal cracks were observed in the matrix and large agglomerated oxide particles.

SEM micrographs show that the microstructure of control alloy consists of a cellular-dendritic structure as shown in Figure 3a,b. Cellular structures primarily exist at the boundaries of the melt pool, where the thermal gradient is largest during the first stage of melt pool solidification [44]. The thermal gradient has a vector component toward the center of the melt pool where much of the thermal mass is contained, but also a vector component oriented along the build direction, since the cooling rate of the solid conduction outweighs conductive and convective cooling at the free surface of the melt pool [45]. As the thermal gradient decreases while moving toward the melt pool center, there is a shift to dendritic structures [46]. There are many occurrences of epitaxial growth across the melt pool boundaries, with new grains adopting the orientation of the crystals at the boundary. Figure 3b contains a change in the dendrite growth direction from [001] to [010], which is along the thermal gradient direction toward the melt pool center; after the crystals propagated for 5–10 µm, they then shifted back to [001] growth direction. Due to the complex heat flow caused by laser scan overlap, raster direction rotation, and layer remelting, the localized thermal gradient is spatially varied with respect to the examination plane.

The microstructures of Cr_2_O_3_ oxide dispersion alloys manufactured using VED of 67.5 J/mm^3^ are shown in SEM micrographs in Figure 3c,d (the YZ plane) and in Figure 3e,f (the XY plane). It can be seen in Figure 3c that some agglomerated nanoscale oxide powders which existed along the melt pool boundaries presumably drifted during AM by buoyancy and Marangoni convection forces [47]. This phenomenon was observed in many locations in this viewing plane. The agglomerates are on the order of 50 µm in size and contain internal cracks due to thermal contraction stresses from the metal matrix during post-solidification cooling, as reported previously in other LPBF oxide-modified powders [26]. The XY plane view in Figure 3e indicates that these internal oxide cracks can easily propagate into the matrix, leading to widespread cracking in the fabricated oxide dispersion samples. Figure 3f displays delamination of the oxide–matrix interface as well as intercellular/interdendritic fracture in the vicinity of the oxide particle. The presence of the oxide particle and thermal contraction of the matrix results in a stress field surrounding the oxide particle, which is sufficient to initiate fracture in the matrix along the weaker direction, transverse to the directional solidification cells/dendrites.

**Figure 4:**
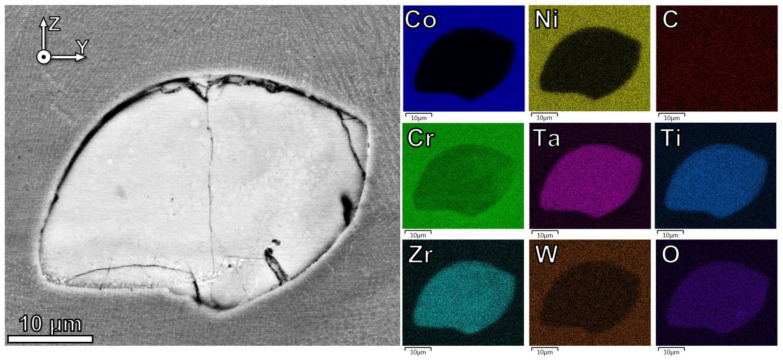
Energy dispersive X-ray spectroscopy (EDS) of a large oxide particle in the ODS alloy, indicating the oxide is enriched with Ta, Ti, Zr, but deficient in Cr.

An intriguing phenomenon that is identified is the reaction of the initial Cr_2_O_3_ oxide into a mixed oxide containing Ta, Ti, and Zr, which are present in the alloy matrix. The enrichment of these elements and the depletion of Cr in oxide clusters is detected by the EDS map in Figure 4. The respective oxides for Ta, Ti, and Zr each have a higher thermodynamic stability/lower Gibbs free energy of formation than the Cr_2_O_3_, implying a net energy gain for these reactions [48]. The reduction–oxidation (redox) reactions governing this change are discussed in Section 4.1.

**Figure 5:**
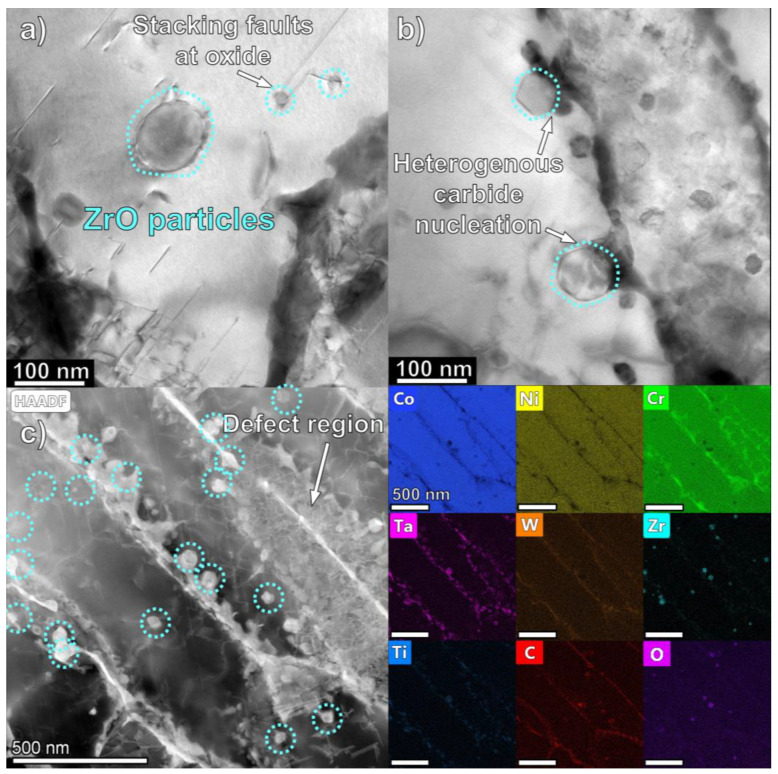
(**a**,**b**) Bright field TEM micrograph of as-printed oxide-modified Mar-M 509 showing nanoscale oxide particles (light contrast) and MC and M_23_C_6_ carbides (dark contrast). (**c**) High-angle angular dark field (HAADF) STEM image and EDS map of cellular regions showing alternating Ta, W, Ti-rich MC, and Cr-rich M_23_C_6_ carbides. Oxide nanoparticles are enriched in Zr and exist both in matrix and coincident with MC carbides.

A TEM micrograph in Figure 5a reveals a second prevalent morphology of oxide particle with a size of 10–100 nm within the matrix. Additional TEM micrographs are available in Appendix A. Stacking faults were observed near oxide particles, where interfacial stresses are at a maximum. Figure 5b shows 80 nm oxide particles with hexagonal morphology sharing an interface with the intercellular carbide precipitates. STEM micrograph and EDS maps in Figure 5c identifies these oxide nanoparticles to be enriched in Zr. Isolated Zr oxides are located within cells but also coincide with some of the MC carbides in the intercellular region.

### 3.3. Mechanical Properties

**Figure 6:**
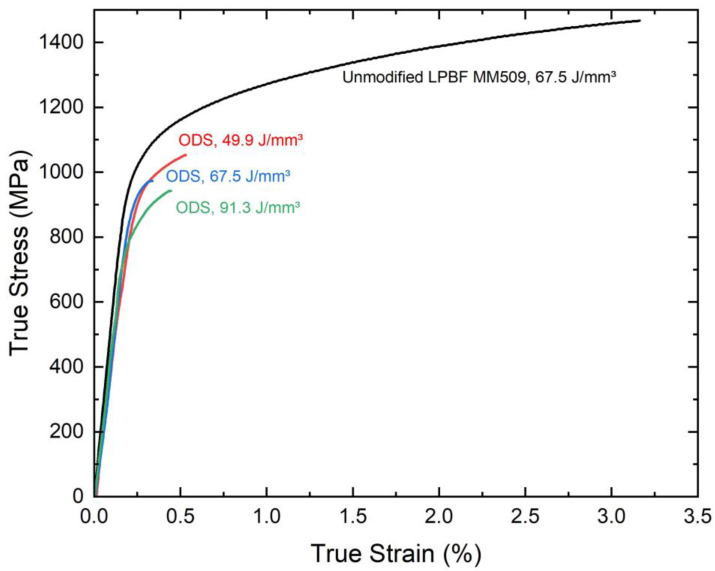
Tensile true stress–strain behavior of control and ODS samples with various energy density. The ODS alloys have reduced strength and tensile ductility.

The plots of room temperature true stress–true strain curves are presented in Figure 6. Regardless of LPBF parameters used in the oxide-modified Mar-M 509, a clear decrease in strength and ductility is observed compared to the control LPBF samples. The calculated yield strength, ultimate tensile strength, and elongation at fracture for each of these curves are given in Table 3. Neither materials exhibited necking behavior prior to fracture.

**Figure 7:**
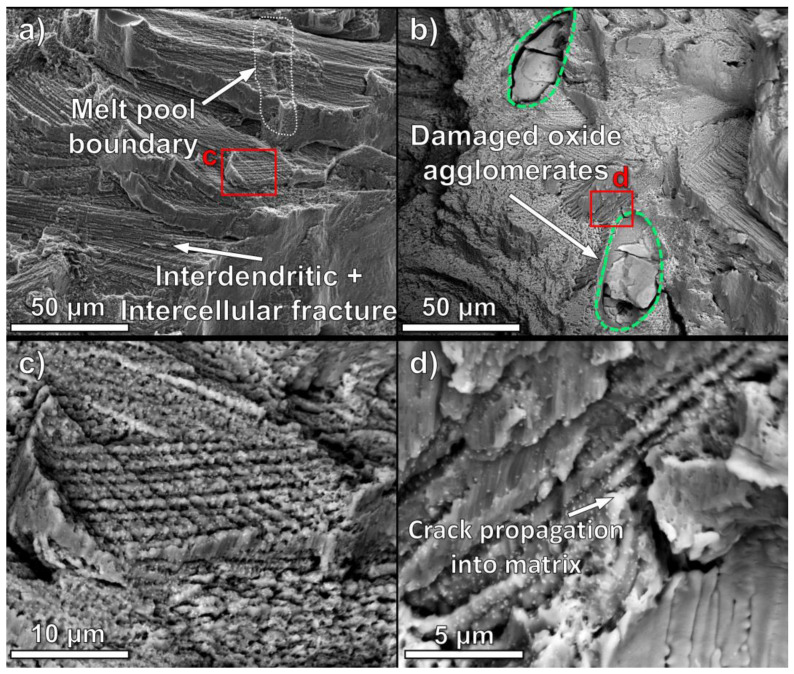
Fracture surface morphology of ODS samples showing (**a**) typical interdendritic and intercellular fracture surfaces and (**b**) cracks originating in oxide particles and propagating into the metal matrix on the fracture surface. High magnification SEM images of the box regions from (**a**,**b**) in (**c**,**d**) display carbides on the interdendritic and intercellular fracture surfaces, indicating that these pathways have reduced resistance to crack propagation.

The fracture surfaces imaged by SEM in Figure 7 inform the cause of reduced strength and ductility in the oxide dispersion materials. Most of the oxide-modified fracture surface consists of brittle intercellular or interdendritic fracture morphology, wherein cracks propagate along the region between the respective directional solidification structures. Cellular regions which coincide with the melt pool boundaries can be distinguished, as shown in Figure 7a. A higher magnification of the interdendritic fracture surface morphology is shown in Figure 7c, with bright nanoscale MC carbides decorating the interdendritic surfaces.

Unlike the control Mar-M509 material, the fracture surfaces of the oxide-modified LPBF material additionally contain 50–100 μm oxide agglomerates, which are internally cracked, and their surfaces are delaminated from the matrix, as can be observed in Figure 7b. In the higher magnification image in Figure 7d, a crack originating at the agglomerate–matrix interface can be observed, propagating into the surrounding matrix.

## 4. Discussion

### 4.1. In Situ Reduction–Oxidation Reactions in LPBF ODS Mar-M 509

The crystal structure of the mixed oxide phase in this work is unconfirmed, and it appears that the large, agglomerated oxides and nanoscale oxides have differing compositions. The fact that the large oxide agglomerates contain Ta, Ti, and Zr and the nanoscale oxides contain primarily Zr suggests that the respective free energies and diffusion distances achievable during LPBF impact the formation mechanism. We have recently reported on a similar Cr_2_O_3_ oxide transformation phenomenon in LPBF Inconel 718 Ni alloy in which the Cr_2_O_3_ undergoes a reduction–oxidation reaction during laser exposure, resulting in the formation of a higher stability of Al_2_O_3_ and TiO_2_ nanoparticles, and these reactions were shown to be exothermic for either pure oxide [49]. Other works utilizing Y_2_O_3_ as the initial oxide in in situ ODS manufacturing have reported the formation of a Y–Al–O oxide, even though the Gibbs free energy of formation at 1480 K for Y_2_O_3_ is lower (−988.5 KJ/mol) than for Al_2_O_3_ (−802.2 KJ/mol), suggesting that the mixed oxide form has an even lower Gibbs free energy of formation than either pure compound [14,48]. Zhang et al. observed the decomposition of Y_2_O_3_ in Co–15Y_2_O_3_ ball-smilled mixtures with increasing milling time and argued that the increase in interfacial energy provides a driving force for decomposition into unstable Y and O, which formed stable Hf, Ti, Zr, and Ca mixed oxides with varying degrees of particle size refinement [27].

Backman and Opila assessed the formation energy and oxygen diffusivity of group IV, V, and VI transition metal oxides and carbides [50]. They found that the group IV elements (Ti and Zr in Mar-M 509) have the most stable oxides with the highest melting temperatures, followed by group V (Ta in Mar-M 509), and lastly, group VI (W in Mar-M 509). Additionally, the oxygen diffusivity was found to be high in zirconia (ZrO_2_), which has the highest melting temperature of the investigated oxides, indicating ZrO_2_ can readily form in the presence of oxygen in the melt pool [50]. These thermodynamic values agree with the observations in this work. It is hypothesized the small 10–100 nm oxide nanoparticles in the presence of ODS Mar-M509 have shorter solidification times and therefore less diffusion, favoring the formation of ZrO_2_. In larger agglomerated particles, there is a higher oxygen concentration and a longer solidification time, so Ti and Ta can diffuse into the oxide, as observed in Figure 4. Tungsten oxides have higher Gibbs free energy of formation and relatively lower solidification temperatures, so Tungsten is not found in the agglomerates or nanoscale oxides. The kinetics of formation of mixed oxides are not well understood, and fundamental research in this area would assist in explaining the size-dependent composition of the oxides found in this material.

### 4.2. Oxide Particle Impact on Microstructure

**Figure 8:**
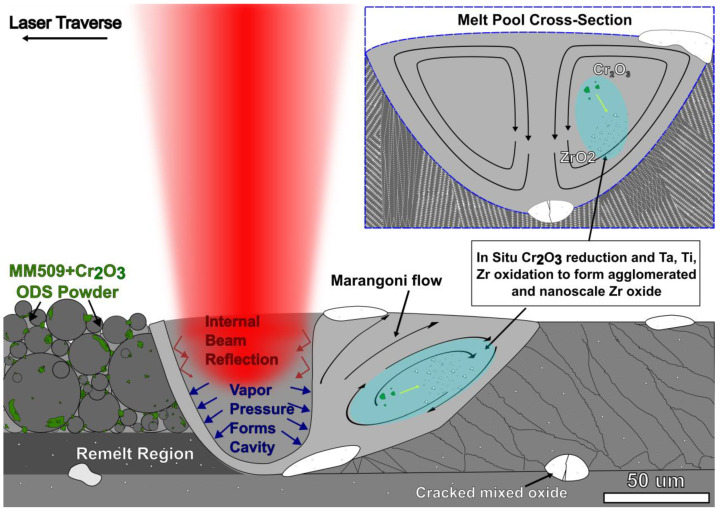
Schematic of in situ oxide reaction in the melt pool, converting Cr_2_O_3_ into a mixed oxide containing Ta, Ti, and Zr. This oxide is present in a submicron spherical form and in an irregular agglomerated form, which is pinned at the melt pool boundary by Marangoni convection and buoyant forces.

A schematic for the proposed mechanism for the evolution of oxide size and positioning within the fluid melt pool is provided in Figure 8. During AM, a melt pool forms. The vaporization of metals creates pressure that maintains a vapor cavity just below the laser spot as the melt pool moves along the track line [51]. As the vaporized metal is expelled upwards from the cavity, gas flows into the resulting lateral low-pressure areas and powder particles are entrained in the gas flow and fall into the melt pool, creating a so-called “denudation zone” [52,53]. Marangoni convective flow within the liquid metal pool distributes nanoscale oxide powders, which solidify above the metal alloy liquidus temperature [51]. Buoyancy forces on the lower-density oxides in combination with circular convection currents causes the pushing of large oxide agglomerates to the melt pool boundaries, both to the surface and the solid–liquid interface, where they are trapped [47,54]. The same distribution of agglomerated oxides at melt pool boundaries has been observed in our single-layer exposure experiments (not shown here) with well-defined boundaries, compared to bulk samples in which some material is remelted multiple times. X-ray diffraction showing reduced crystallographic texture may suggest that the presence of oxide particles provides additional nucleation sites, which result in more equiaxed grain structure [55].

From the TEM micrograph in Figure 5, it is evident that oxides in the 10–100 nm range also exist within the microstructure. The exact formation mechanism of these smaller particles is unconfirmed, although due to their high melting temperature, the oxide particles solidify before the Co matrix. It is hypothesized that either the turbulent Marangoni flow, the reduction–oxidation reaction, or some combination of these effects is responsible for the formation of nanoscale oxides. The interdendritic/intercellular continuous carbide chains appear to nucleate heterogeneously on the oxide particle surfaces, as seen in Figure 5b. Mao et al. found a semi-coherent relationship between Y_2_Ti_2_O_7_ and an austenitic steel matrix, specifically ${\left(\bar{2}20\right)}_{Y2Ti2O7}||{\left(200\right)}_{Matrix}$ and ${\left(\bar{3}\bar{3}1\right)}_{Y2Ti2O7}||{\left(0\bar{2}2\right)}_{Matrix}$ orientation relationships [56]. Another possible mechanism is the formation of zirconium oxide using the MC carbides as a source of zirconium, since ZrO has a lower Gibbs free energy and the oxidation rate of ZrC is 2× higher than TaC and over 10× higher than TiC [50,57].

### 4.3. Oxide Particle Impact on Mechanical Properties

Due to the limited strain tolerance of the directionally solidified matrix–carbide structures found in LPBF Mar-M 509, the stress fields around the large oxide agglomerates cannot be accommodated with plasticity, leading to cracking. The formation of Zr-rich oxide nanoparticles suggests the potential to achieve a stable, Orowan-strengthened ODS microstructure. The stress fields generated by the nanoscale oxides are less impactful and are shown to be accommodated by the generation of stacking faults near the oxide/matrix interface, as can be seen in Figure 5. In an idealized microstructure without large agglomerates and cracks, nanoscale oxide-particle-induced planar defect generation may further inhibit dislocation motion, providing additional strength.

## 5. Conclusions

This work investigated the formation and dispersion of oxides through the LPBF of Mar-M 509 and Cr_2_O_3_ powders. The following conclusions are made:

1. A reduction–oxidation transformation from Cr_2_O_3_ to a more stable mixed tantalum, titanium, and zirconium oxide is observed. These reactions could be used to achieve higher entropy mixed oxide compositions in situ.
2. The macroscale brittle behavior of oxide dispersion of Mar-M 509 is caused by cracking near the 50–100 µm agglomerated oxide particles. Residual stress-induced cracks in the oxide particles propagate into the matrix and rapidly grow along the aligned carbide precipitates in the as-printed Mar-M 509.
3. Zirconium-rich nanoscale oxides in the matrix provide potential for an idealized oxide-dispersion-strengthened system for high temperature applications due to the high thermal stability of the oxide particles.

## Figures and Tables

**Table 1:** Composition of laser powder bed fusion (LPBF) Mar M-509 powder as provided by PST, in weight percent.

Co	Cr	Ni	C	W	Ta	Ti	Zr	Fe	Si
Bal.	23.82	10.19	0.60	6.98	3.37	0.23	0.45	0.02	0.07

**Table 2:** Laser-powder-bed-fusion parameters for examined samples.

Parameters	Energy Density (J/mm^3^)	Laser Power (W)	Scan Speed (mm/s)	Hatch Spacing (µm)	Layer Thickness (µm)
Set 1	49.9	242	1104	110	40
Set 2	67.5	285	960	110	40
Set 3	91.3	328	816	110	40

**Table 3:** Tensile performance of ODS samples with varying energy density produced by LPBF compared to baseline powder, indicating premature fracture in ODS samples.

Sample	Yield Strength (MPa)	Ultimate Tensile Strength (MPa)	Elongation at Fracture (%)
Control 67 J/mm^3^	954	1420	3.2
ODS 49 J/mm^3^	797	1054	0.55
ODS 67 J/mm^3^	756	973	0.34
ODS 91 J/mm^3^	695	942	0.54

**Figure A1:**
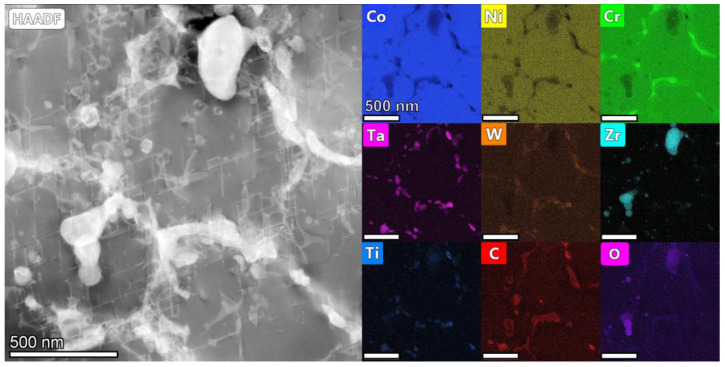
HAADF STEM micrograph and EDS maps of a cellular region, with the cell elongation direction out of plane and a hexagonal cross-section. Carbide and oxide precipitates exist in the intercellular boundaries and some Zr oxide is located within the cells.

## Data Availability

Not applicable.
